# Interventional diagnostic procedures in INOCA: an essential approach in ischemic heart disease

**DOI:** 10.1007/s12928-025-01197-4

**Published:** 2025-11-02

**Authors:** Yasushi Matsuzawa, Masanobu Ishii, Hiroaki Kusaka, Takayoshi Yamashita, Eiichiro Yamamoto, Kenichi Tsujita

**Affiliations:** Department of Cardiovascular Medicine, Graduate School of Medical Sciences, Kumamoto University, 1-1-1 Honjo, Chuo-Ku, Kumamoto, 860-8556 Japan

**Keywords:** Ischemia with non-obstructive coronary arteries, Coronary microvascular dysfunction, Interventional diagnostic procedures, Coronary vasospasm, Precision cardiovascular medicine

## Abstract

Approximately 60% of patients with angina symptoms do not have obstructive coronary lesions, a condition known as ischemia with non-obstructive coronary arteries (INOCA). Among these patients, coronary microvascular dysfunction (CMD) is found in nearly half. The epicardial coronary arteries represent only a small portion of the heart’s vascular bed, with CMD increasingly recognized as a significant mechanism of myocardial ischemia in INOCA. Recent revisions in the Japanese and European guidelines emphasize the importance of a comprehensive functional evaluation through interventional diagnostic procedure (IDP) for diagnosing CMD and coronary vasospastic angina, two primary causes of ischemia in INOCA patients. IDP allows clinicians to identify the underlying endotype and implement tailored therapeutic strategies, moving beyond empirical therapy. Despite its clinical relevance, INOCA remains under-recognized due to a lack of awareness among healthcare providers as well as the general public, leading to diagnostic delays and undertreatment. Public education campaigns and clinician training are crucial for improving disease recognition and reducing diagnostic delay. Future directions for INOCA management include standardizing and simplifying IDP protocols, incorporating artificial intelligence for diagnostic support, and developing non-invasive alternatives for coronary functional testing. These efforts will enhance the accuracy and accessibility of IDP, facilitating its integration into routine clinical practice. Ultimately, the continued evolution of IDP will play a key role in advancing precision cardiovascular medicine, bridging the gap between symptoms, diagnosis, and meaningful care, and improving outcomes for INOCA patients.

## Introduction

**Fig. 1:**
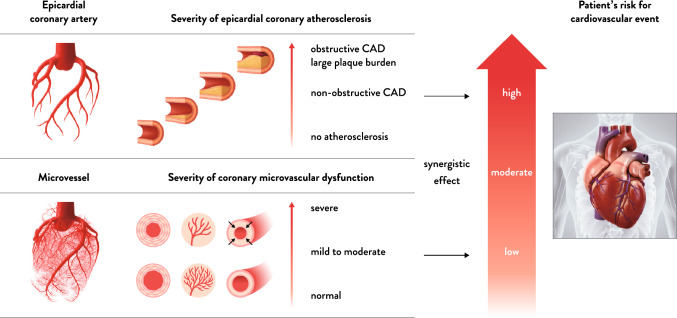
Synergistic Impact of Coronary Microvasculature and Epicardial Coronary Arteries on Patient Outcomes. The epicardial coronary arteries represent only a small portion of the heart’s vascular bed. The interplay between coronary microvascular dysfunction and epicardial coronary atherosclerosis influences cardiovascular outcomes [11]. Abbreviations: *CAD* coronary artery disease

Approximately 60% of patients with angina symptoms do not have obstructive coronary lesions—a condition referred to as ischemia with non-obstructive coronary arteries (INOCA). Among these patients, about half exhibit coronary microvascular dysfunction (CMD) [1, 2]. The epicardial coronary arteries represent only a small portion of the heart’s vascular bed (Fig. 1), and coronary microvascular dysfunction is increasingly recognized as an important mechanism of myocardial ischemia in patients with INOCA. In the 2023 revision of the Japanese guidelines, invasive functional assessment was given a Class IIa recommendation with a Level of Evidence B [3]. In 2024, the European Society of Cardiology guidelines were updated, and invasive functional assessment was upgraded to a Class I recommendation with a Level of Evidence B [4]. Both guidelines emphasize the importance of diagnosing coronary vasospastic angina and microvascular angina, which are two major causes of myocardial ischemia. They recommend a comprehensive diagnostic approach that combines coronary spasm provocation testing with functional assessment of the coronary microcirculation. These conditions frequently coexist, and it is crucial to determine the endotype through an interventional diagnostic procedure (IDP) to guide appropriate therapy. Without proper diagnostic evaluation, coronary vasospasm and microvascular dysfunction may go undetected [5], potentially leading to an increased risk of cardiovascular events. Despite its clinical relevance, INOCA remains under-recognized not only among the general public but also within clinicians. This lack of awareness can result in delayed diagnoses and undertreatment. Therefore, public education efforts are essential to improve disease recognition, encourage appropriate healthcare-seeking behavior, and promote the adoption of evidence-based diagnostic approaches such as IDP [6]. It is expected that the diagnosis of INOCA will allow for identification of the underlying endotype, and that providing endotype-specific treatment will reduce the risk of cardiovascular events by clarifying the presence of disease. However, IDPs are not yet widely adopted, due to factors such as extended procedure time, potential procedural risks, and the absence of reimbursement. Beyond these concerns regarding complexity and risk, a proper understanding of the clinical value and patient benefits of IDP is essential for its appropriate implementation and broader adoption.

### Definition and prognosis of INOCA

INOCA is defined by three criteria: [3]

(i) Stable, chronic chest symptoms (including cardiac-like and non-cardiac-like symtoms).
(ii) (ii) Objective evidence of myocardial ischemia. "Such as electrocardiography, echocardiography, cardiac magnetic resonance imaging, nuclear imaging, or invasive cardiac catheterization."
(iii) No significant coronary artery stenosis—defined as the absence of obstructive coronary artery disease (CAD) with ≥ 50% luminal narrowing on coronary angiography or coronary computed tomography angiography, and no functionally significant ischemia, such as a fractional flow reserve (FFR) ≤ 0.80.

Patients with coronary artery spasm (CAS) and CMD, the two major causes of INOCA, have a worse prognosis compared to those without these conditions [7, 8]. CAS is associated with a variety of arrhythmias, including sinus bradycardia, complete atrioventricular block, paroxysmal atrial fibrillation, premature ventricular contractions, ventricular tachycardia, ventricular fibrillation, and asystole. These life-threatening arrhythmias often occur in the setting of CAS-related acute coronary syndrome [9] and thus CAS has been linked to increased risks of all-cause mortality and cardiac death. On the other hand, when CMD is defined by a reduced coronary flow reserve (CFR), patients with CMD have been reported to exhibit approximately a fourfold increase in all-cause mortality (hazard ratio: 3.93, 95% confidence interval (CI) 2.91–5.30, *P* < 0.001) and a fivefold increase in the incidence of major adverse cardiac events (MACE) (odds ratio: 5.16, 95% CI 2.81–9.47, *P* < 0.001), compared to those without CMD [10]. Patient prognosis is determined by the interplay between the severity of CMD and the extent of epicardial coronary atherosclerosis (Fig. 1). [11] Therefore, identifying these two major endotypes of INOCA allows for the recognition of high-risk patients who may benefit substantially from targeted therapy aimed at preventing serious cardiovascular events, including sudden death.

### Prevalence and endotype proportions

**Fig. 2:**
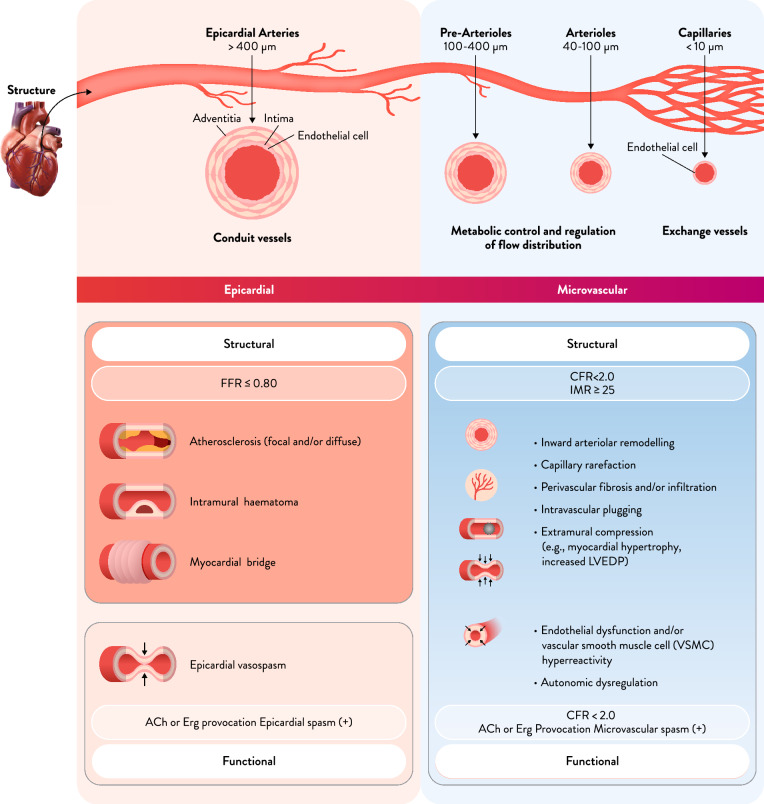
Structural and Functional Abnormalities of the Epicardial Coronary Arteries and the Coronary Microvasculature. The coronary arterial system is anatomically and functionally divided into epicardial arteries, pre-arterioles, arterioles, and capillaries. The latter three constitute the coronary microcirculation. Structural abnormalities of the epicardial arteries are exemplified by atherosclerotic plaque formation, whereas functional abnormalities include epicardial coronary artery spasm. Pre-arterioles and arterioles act as resistance vessels, and their dysfunction—whether due to impaired endothelial and/or smooth muscle function—can lead to reduced vasodilatory capacity and microvascular spasm. Structural alterations in the microcirculation include inward arteriolar remodeling, capillary rarefaction, intravascular plugging, and perivascular fibrosis, all of which contribute to coronary microvascular dysfunction. Abbreviations: *ACh* acetylcholine, *CFR* coronary flow reserve, *Erg* ergonovine, *FFR* fractional flow reserve, *IMR* index of microcirculatory resistance, *LVEDP* left ventricular end-diastolic pressure

A wide range of structural and functional abnormalities can be found from the epicardial coronary arteries to the microvasculature, and ischemic heart disease can be classified into distinct endotypes based on the underlying pathophysiological mechanisms (Fig. 2). A systematic review and meta-analysis of 56 studies involving 14,427 patients with INOCA was conducted to determine the prevalence of CMD and coronary vasospasm [12]. The pooled prevalence of CMD was 41% (95% CI 36–47%), epicardial vasospasm was 40% (95% CI 34–46%), and microvascular spasm was 24% (95% CI 21–28%). Furthermore, 23% (95% CI 17–31%) of patients had both CMD and vasospastic angina, indicating a significant overlap. These findings suggest that relying on a single diagnostic modality may lead to misclassification of the endotype in approximately one-quarter of cases.

Although these results provide important insights, the majority of the included studies were conducted in Europe, and prevalence rates may differ in other populations. Therefore, there is a need to establish region-specific evidence, particularly for clinical practice in Japan.

## Diagnostic approach and practical criteria for INOCA

### Integrated testing of coronary spasm and CMD: unresolved issues in procedural sequencing

**Fig. 3:**
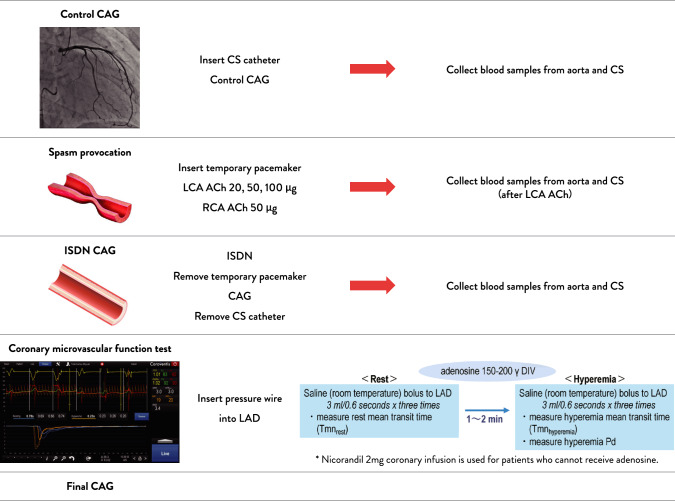
Protocol of the Comprehensive Interventional Diagnostic Procedure at Kumamoto University Hospital. A sheath was inserted into the radial artery, and two sheaths were placed in the femoral vein. Through one of the femoral venous sheaths, a CS catheter was advanced. A catheter was engaged in the RCA, and blood samples were collected from both the aorta and the CS. Following control CAG, a temporary pacemaker was placed, and an ACh provocation test was performed. After administering the maximal dose of ACh into the LCA, blood samples were again collected from the aorta and the CS. Upon completion of the provocation test, intracoronary ISDN was administered, and resolution was confirmed angiographically before removing the temporary pacemaker. After ISDN administration, CAG was repeated, and final blood samples were obtained from both the aorta and the CS. The CS catheter was then removed. A pressure wire was advanced into the LAD to assess CFR and the IMR. After withdrawal of the pressure wire, a final angiogram was performed to evaluate for procedural complications such as coronary dissection or wire perforation. The procedure was concluded upon confirmation of the absence of such complications. Abbreviations: *ACh* acetylcholine, *CAG* coronary angiography, *CFR* coronary flow reserve, CS coronary sinus, IMR index of microcirculatory resistance, *ISDN* isosorbide dinitrate, *LAD* left anterior descending artery, *LCA* left coronary artery, *LVEDP* left ventricular end-diastolic pressure, *RCA* right coronary artery

The guidelines recommend that coronary spasm provocation testing using acetylcholine or ergonovine, as well as coronary microcirculatory assessments such as CFR and the index of microcirculatory resistance (IMR) using vasodilators, be performed during the same session as coronary angiography. The protocol of our institution is presented as an example, and standardization of procedures including such protocols is desired (Fig. 3).

Currently, there is insufficient evidence to determine the optimal sequence in which these tests should be performed. Two potential concerns have been raised:

(i) If the adenosine stress test is performed first:
  The preceding administration of vasodilators may reduce the likelihood of coronary spasm induction, potentially leading to false-negative results for coronary vasospasm.
(ii) (ii) If the acetylcholine stress test is performed first:
  Residual microvascular spasm may impair subsequent vasodilation, potentially resulting in false-positive findings for CMD.

Previous studies have reported that epicardial vasospasm is not provoked in approximately one-quarter of patients undergoing acetylcholine testing following nitroglycerin administration, whereas microvascular spasm persists in approximately 80% of patients despite nitroglycerin [13]. These findings highlight an important area for future research.

### Diagnostic criteria for coronary spasm

In coronary spasm provocation testing using acetylcholine or ergonovine, angiographic findings indicative of epicardial coronary spasm are defined as either (1) transient complete or subcomplete occlusion (> 90% stenosis) localized to a single coronary segment, or (2) 90% diffuse vasoconstriction involving two or more contiguous coronary segments, accompanied by evidence of myocardial ischemia (i.e., angina pectoris and ischemic electrocardiographic changes).

In the absence of coronary spasm testing, suspected coronary spasm may be diagnosed based on angina-like episodes that resolve promptly with nitrates, along with at least one of the following clinical features:

(i) Occurs at rest, particularly during nighttime or early morning hours.
(ii) Exhibits marked diurnal variation in exercise tolerance (especially reduced tolerance in the early morning).
(iii) Provoked by hyperventilation.
(iv) Responsive to calcium channel blockers but not to β-blockers.

Positive findings for microvascular spasm are defined as the presence of myocardial ischemia (angina and ischemic electrocardiographic changes) in the absence of significant epicardial coronary artery stenosis or occlusion. However, this diagnostic approach does not directly detect microvascular spasm. In such cases, biochemical assessment of ischemia—such as elevated lactate levels in the coronary sinus—may serve as an additional diagnostic tool.

### Diagnostic criteria for coronary microvascular dysfunction (Fig. 2)

Microvessels in the coronary microcirculation are difficult to visualize directly via angiography. Therefore, the evaluation of coronary microcirculation typically relies on mean transit time (Tmn), which is a surrogate marker of pressure and blood flow velocity, measured using a PressureWire (Abbott Vascular). The procedure follows the conventional approach for FFR measurement. A sensor is delivered to the target vessel, and saline is injected into the coronary artery three times—at rest and during maximum hyperemia—to measure both pressure and Tmn (Fig. 3).

In contemporary clinical practice, CFR and IMR represent the most commonly applied modalities for evaluating the coronary microcirculation. The current Japanese guidelines adopt a cut-off value of CFR < 2.0 and IMR ≥ 25 for the diagnosis of positive CMD [3]. Until 2024, both the Japanese and European guidelines unified the cut-off value for CFR at < 2.0 for a positive CMD diagnosis [3, 14]. However, in the 2024 revision of the European guidelines, the CFR cut-off value was raised to < 2.5 [4]. This change has resulted in a discrepancy between the two guidelines. The recently proposed CATH CMD algorithm designates CFR values between 2.0 and 2.4 as a “gray zone.” [15] This suggests that diagnosing CMD should not rely solely on a single cut-off value but also take this intermediate range into account. In contrast, the cut-off value for IMR as a positive diagnosis of CMD remains consistent across both guidelines, with a threshold of ≥ 25.

## Clinical benefits and potential risks of IDP: when to use and when to avoid

### Benefits

Careful and thorough history taking is the cornerstone of the initial diagnostic approach to INOCA. Compared to obstructive CAD, patients with INOCA often lack definitive clinical findings apart from their history, making detailed history taking particularly crucial. For obstructive CAD, clinical tools such as symptom scores and the number of CAD risk factors are used to estimate the Risk Factor–weighted Clinical Likelihood of obstructive disease. However, this kind of stratification is not easily applicable to INOCA. When history, physical examination, and basic tests fail to identify a non-cardiac cause for symptoms, further evaluation with a suspicion for INOCA should be pursued.

**Fig. 4:**
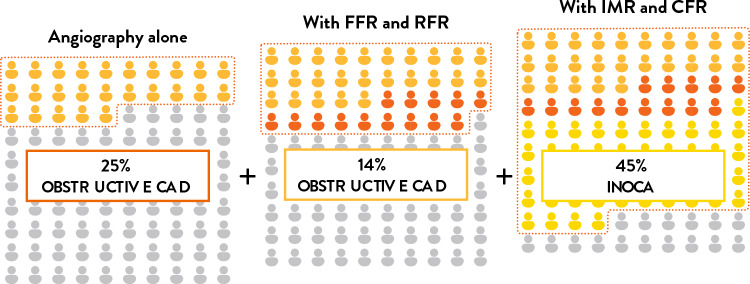
Overview of the Advanced Invasive Diagnosis (AID)-Angio Study. Results from the AID-Angio study indicate that combining angiography with intracoronary diagnostic testing significantly improves diagnostic accuracy in chronic coronary syndromes

In addition, while many patients with obstructive CAD do not present with “typical” angina, this is even more frequently the case in CMD and coronary vasospasm, where symptoms often deviate from classical descriptions of angina, further complicating diagnosis. Importantly, the PRECISE study showed that patients with “typical” and “atypical” angina had comparable 1-year outcomes [16], underscoring the limited prognostic utility of symptom classification based solely on angina typicality, as used in obstructive CAD prediction models. Therefore, it is essential to carefully evaluate chest pain and objectively exclude myocardial ischemia due to obstructive CAD, microvascular dysfunction, or coronary vasospasm before concluding that the symptoms are of non-cardiac origin. The Advanced Invasive Diagnosis (AID)-Angio study [17], presented at EuroPCR 2024 (Fig. 4). When evaluating coronary abnormalities responsible for ischaemia, the predefined application of the AID strategy resulted in a 2.6-fold higher diagnostic yield than invasive coronary angiography alone (84.2% vs 32.2%), primarily driven by improved detection of INOCA. The study also highlighted the following key findings: (1) compared to angiography alone, 59% more patients were accurately diagnosed with ischemia using the AID strategy, emphasizing that angiography alone is insufficient for ischemia diagnosis; (2) only 39% of patients had obstructive epicardial CAD, while 45% had INOCA, with INOCA being more prevalent than obstructive epicardial CAD; and (3) approximately 60% of patients had their initial treatment plan modified based on the IDP results. These findings suggest that a comprehensive functional evaluation through IDP can reduce the number of undiagnosed patients, thus enabling more appropriate treatment for a greater number of patients.

**Fig. 5:**
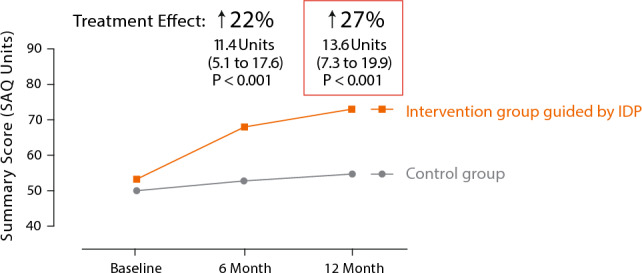
Sustained Benefits of an IDP-Guided Strategy on Angina and Quality of Life: Insights from the CorMiCa Trial. The implementation of IDP resulted in a 22% and 27% higher Seattle Angina Questionnaire score at 6 months and 1 year, respectively, in the intervention group compared to the control group. Abbreviations: *IDP* interventional diagnostic procedure

The CorMiCA trial compared the outcomes, including quality of life (QOL) and MACE, of 151 patients with INOCA between the intervention group (n = 76), which received drug therapy guided by IDP results, and the control group (*n* = 75), which received general treatment based on imaging findings [18]. The results showed that the intervention group, guided by IDP, was able to select treatment plans more aligned with clinical guidelines. Furthermore, the Seattle Angina Questionnaire (SAQ) score at 1 year was 27% higher (13.6 points, 95% CI 7.3–19.9, *P* < 0.001) in the intervention group compared to the control group (Fig. 5). These results suggest that IDP contributed to significant improvements in angina symptoms and QOL, with sustained benefits over the 1-year period. Importantly, the effects of IDP are not temporary but ongoing.

### Potential risks

Invasive coronary angiography using radial artery access is considered to have a low risk of complications. However, when conducting a coronary spasm provocation test, especially in patients with high spasm activity or multivessel spasm, there is a risk of inducing severe and widespread coronary spasm. This could potentially lead to hypotension, cardiogenic shock, life-threatening arrhythmias, or even cardiac arrest. Therefore, it is critical to have a well-trained expert team for these procedures to minimize the risk of complications. In the event of induced spasm, immediate interventions such as intracoronary administration of nitroglycerin to relieve the spasm, administration of vasopressors to address hypotension, and prompt management of serious arrhythmias are essential. Recent studies have shown that during acetylcholine and ergonovine provocation tests, serious complications occurred in approximately 0.89% of the 17,700 cases, with one death (0.006%) and two cases of acute myocardial infarction (0.01%) [19]. To reduce the risk of complications, it is essential to follow a well-standardized procedure and carefully select the appropriate protocol. Additionally, it is recommended that these tests be performed by an expert team who are familiar with the procedure. In acute coronary syndrome, careful consideration should be given to whether or not to proceed with the test. Only after ensuring the exclusion of other conditions, such as plaque rupture or spontaneous coronary artery dissection, and taking appropriate safety precautions, should the test be conducted. This approach has been emphasized in updated guidelines, which reflect a more cautious and standardized approach to performing these provocative tests [3, 20].

### Therapeutic approaches tailored to specific endotypes of INOCA 

**Fig. 6:**
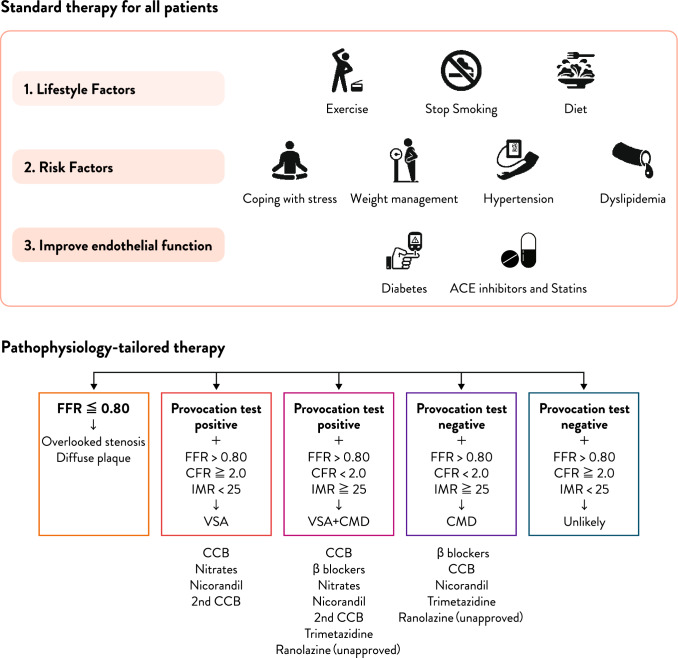
A holistic, patient-centered approach to INOCA management. Managing INOCA is challenging due to its heterogeneous mechanisms. IDP allow for detailed phenotyping, enabling tailored therapy based on underlying pathophysiology. This precision-medicine approach improves symptom control and quality of life. Spasm and microvascular dysfunction can be effectively treated when appropriately identified. Abbreviations: *IDP* invasive diagnostic procedures, *INOCA* ischemia with non-obstructive coronary arteries

Managing anginal symptoms in patients with INOCA remains difficult due to the heterogeneity of this population and the absence of robust randomized clinical trials. Phenotyping of INOCA enables the application of tailored therapy by aligning treatment strategies with the underlying pathophysiological mechanisms, thereby improving patient outcomes. INOCA encompasses several distinct endotypes that are often undetectable by standard coronary imaging but can be identified through invasive coronary function testing. Each phenotype offers clinically actionable insights. For instance, epicardial and microvascular spasm often respond well to calcium channel blockers and nitrates. In contrast, CMD diagnosed by reduced CFR and/or elevated IMR is generally treated with β-blockers as first-line therapy. These patients frequently present with endothelial dysfunction or non-obstructive atherosclerosis, for which lifestyle modifications—including exercise, dietary control, smoking cessation, and weight reduction—together with statins and angiotensin-converting enzyme inhibitors, have been shown to be effective through their beneficial effects on endothelial function. In certain patients, endothelial dysfunction may arise from different underlying mechanisms, including sleep disturbances, psychological stress, and various forms of chronic inflammation. Accordingly, clinicians should be mindful of these non-conventional factors in addition to the usual determinants. In the management of patients, non-invasive evaluation of vascular endothelial function provides clinical utility. Moreover, the coexistence of spasm and CMD is not uncommon. Although β-blockers are typically avoided in vasospastic angina due to potential exacerbation of spasm, when the pathophysiology is clearly delineated, a combination of β-blockers and calcium channel blockers may be rationally used in selected cases (Fig. 6).

This precision-medicine approach moves beyond the traditional "one-size-fits-all" model, allowing for individualized care based on the predominant mechanism of ischemia. Importantly, the IDP provides a safe and efficient means to evaluate these functional domains within a single session, often immediately after coronary angiography. Tailored pharmacological treatment based on IDP findings has been shown to significantly improve symptom control and quality of life in patients with INOCA [18]. As cardiovascular care continues to evolve toward a more personalized model, routine functional assessment of coronary abnormalities in patients with angina and non-obstructive coronary arteries should be considered an essential component of evidence-based clinical decision-making.

Although pharmacological therapy based on IDP findings has shown substantial benefits, managing patients with INOCA requires a more holistic and individualized strategy. Because coronary atherosclerosis and endothelial dysfunction are frequently observed in these patients, it is important to provide tailored guidance that encourages healthy lifestyle changes [4]. These efforts should aim not only to address conventional cardiovascular risk factors, but also to support vascular health, relieve symptoms, and improve both long-term outcomes and daily well-being. Accordingly, lifestyle optimization and comprehensive risk management form the foundation of care for all individuals diagnosed with INOCA.

### Device aided therapy for INOCA

Recently developed coronary sinus reducer (CSR) has been reported to improve myocardial perfusion and reduce angina symptoms in patients with stable coronary artery disease [21]. Although the efficacy of this device in patients with INOCA is unknown, there is hope for the treatment of patients with CMD. The target of treatment using CSR is limited to relatively severe patients who are judged to have no treatment options other than CSR. It may be possible to stratify the severity using CFR and IMR values and apply CSR treatment to more severe cases effectively.

### Future directions and research priorities

Despite recent advances in the clinical application of IDP, several areas remain open for future investigation and development.

### Standardization and simplification of IDP procedures

There is a growing need to establish standardized protocols and simplify IDP techniques to ensure broader adoption in routine clinical practice. Streamlining procedural steps, reducing procedure time, and improving reproducibility across centers will be essential to promote widespread use, particularly in non-tertiary care settings.

### Generation of domestic evidence and cross-regional comparisons

To ensure the applicability of IDP-based strategies across diverse populations, accumulating clinical evidence within Japan is critical. Comparative studies with Western cohorts may elucidate potential ethnic, environmental, and healthcare system-related differences in disease characteristics and treatment responses.

### Integration of artificial intelligence and machine learning

The use of artificial intelligence and machine learning holds significant potential for improving diagnostic accuracy and procedural efficiency. Automated interpretation of pressure-flow data, pattern recognition, and endotype classification may support clinical decision-making and reduce inter-operator variability, facilitating broader clinical adoption.

### Development of novel therapeutic strategies

Emerging treatment approaches such as developing other drugs and device-based interventions represent promising options for patients with INOCA who remain symptomatic despite the current medical therapy. Future clinical trials are needed to evaluate these modalities based on specific endotypes identified by IDP.

### Towards non-invasive functional assessment

While invasive testing currently remains the gold standard for coronary functional assessment, the development of accurate non-invasive modalities would be a major advancement. Achieving reliable non-invasive surrogates for invasive measures like CFR, IMR, or vasospasm reactivity would allow wider access to personalized diagnostics and facilitate long-term disease monitoring without procedural burden.

In summary, the continued evolution of IDP and its surrounding ecosystem—including innovation, standardization, and non-invasive alternatives—will be essential to realize precision medicine in patients with angina and no obstructive coronary artery disease.

## Conclusion

IDP have emerged as a cornerstone in the assessment and management of patients with INOCA. By enabling the detailed phenotyping of underlying coronary functional abnormalities—such as epicardial spasm, microvascular spasm, impaired CFR, and elevated IMR—IDP allows clinicians to move beyond empirical therapy and implement truly tailored treatment strategies. These mechanism-specific approaches have been shown to improve symptom control and quality of life, offering hope to a patient population that has long been underserved.

However, the broader impact of IDP-based care requires not only advancements in clinical technique and evidence generation but also an increase in awareness among healthcare providers as well as the general public. Many patients with INOCA remain undiagnosed or misattributed due to a lack of recognition of the disease, even among healthcare providers. Therefore, public education campaigns and community engagement are essential to promote understanding of INOCA, reduce diagnostic delay, and encourage patients to seek appropriate medical evaluation.

Future directions include the standardization and simplification of IDP protocols, the generation of domestic evidence within Japan to complement international data, the integration of artificial inteligence and machine learning for diagnostic support, and the development of less invasive alternatives for coronary functional testing. As these initiatives evolve, IDP will remain a key component in the shift toward precision cardiovascular medicine—bridging the gap between symptoms and diagnosis, and between diagnosis and meaningful care.
